# Synthesis and Crystal Structure of Novel Sulfone Derivatives Containing 1,2,4-Triazole Moieties

**DOI:** 10.3390/molecules15020766

**Published:** 2010-02-03

**Authors:** Weiming Xu, Baoan Song, Pinaki Bhadury, Yang Song, Deyu Hu

**Affiliations:** Key Laboratory of Green Pesticide and Agriculture Bioengineering, Ministry of Education, Research and Development Center for Fine Chemicals, Guizhou University, Guiyang 550025, China; E-Mails: xuweiming2009@163.com (W.X.); bhadury@gzu.edu.cn (P.B.); yangsdqj@gmail.com (Y.S.); fcc.dyhu@gzu.edu.cn (D.H.)

**Keywords:** sulfone derivatives, 1,2,4-triazole, sodium tungstate, hydrogen peroxide, synthesis, crystal structure

## Abstract

Some 3-(Substituted methylthio)-4-phenyl-5-(3,4,5-trimethoxyphenyl)-4*H*-1,2,4-triazole derivatives were synthesized in six steps starting from easily accessible gallic acid. The resulting sulfides were then catalytically oxidized to the title sulfones with H_2_O_2_. The products were obtained in high yield under mild conditions and practically devoid of any by-products. The structures were confirmed by elemental analysis, IR, ^1^H- and ^13^C-NMR spectral data. Furthermore, a detailed X-ray crystallography structural analysis of model triazole **7g** was carried out.

## 1. Introduction

**Scheme 1:**
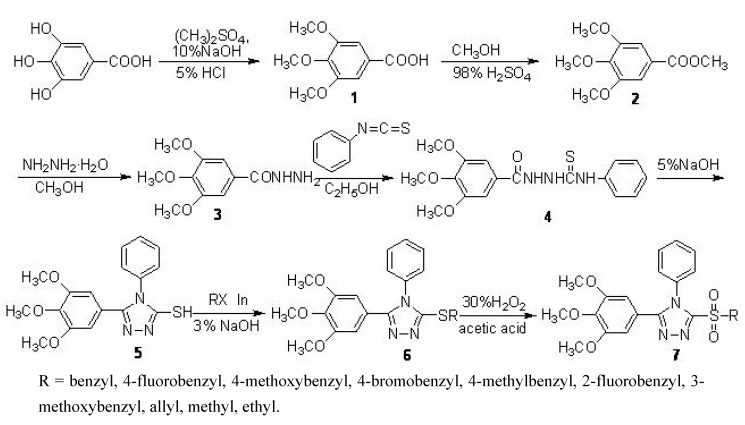
Reagents and conditions: synthetic route to title compounds **6** and **7**.

Organic compounds incorporating heterocyclic ring systems continue to attract considerable interest due to their wide range of biological activities. In this context, heterocyclic compounds bearing 1,2,4-triazole scaffolds find wide application both in medicinal chemistry as antibacterial [1], antimicrobial [2], antidepressant [3], antiinflammatory [4], antiviral [5], and human antifungal [6] agents, and in agricultural science as potent fungicides [7], herbicides [8] and insecticides [9]. A large number of sulfur-containing 1,2,4-triazoles are known for their biological activities [10,11,12] and the corresponding sulfone analogues are encountered in a large number of patents and chemical literature describing extensive commercial applications [13]. Based on our earlier observation that certain appropriately substituted 2-sulfonyl-5-(3,4,5-trimethoxyphenyl)-1,3,4-thiadiazole and 2-sulfonyl-5-(3,4,5-trimethoxyphenyl)-1,3,4-oxadiazole derivatives possessed good bioactivity [14], we undertook the synthesis of some additional triazole derivatives from naturally occurring gallic acid [15], and evaluated them for their bioactivity profile after subtle structural modification. The synthetic routes to the sulfides **6** and title sulfones **7** are depicted in Scheme 1. The structures of all the compounds were verified by the IR, NMR, and elemental analysis. Furthermore, 3-(3-methoxybenzylsulfonyl)-4-phenyl-5-(3,4,5-trimethoxyphenyl)-4*H*-1,2,4-triazole (**7g**) was subjected to detailed investigation by X-ray crystallography.

## 2. Results and Discussion

### 2.1. Chemistry

3,4,5-Trimethoxybenzhydrazide (**3**) was synthesized from the starting material gallic acid through etherification, esterification and hydrazidation. The compound **3** was then converted into **4** by reaction with 1-isothiocyanatobenzene in ethanol. Cyclization of this intermediate with sodium hydroxide under reflux conditions afforded 4-phenyl-5-(3,4,5-trimethoxyphenyl)-4H-1,2,4-triazole-3-thiol (**5**). Subsequently, this triazole analogue was converted to the thioether derivatives **6** in a thioetherification reaction with suitable halides (RX) catalyzed by indium or indium trichloride [16]. Treatment of the sulfides with H_2_O_2_ catalyzed by sodium tungstate finally produced the title heterocyclic sulfone derivatives **7**. Although the electron rich thioether can be oxidized to sulfone by a variety of agents such as *m*-CPBA [17], CH_3_CO_3_H [18], halogen derivatives NaClO [19], H_5_IO_6_ [20], transition metal derivatives e.g., manganese (iv) oxide [21] and KMnO_4_ [22], none of them seem to match the advantages offered by aqueous hydrogen peroxide, which is an ideal environmentally-friendly waste-avoiding oxidant with water being the only theoretical by-product of the reaction. Moreover, due to its high solubility in water and many organic solvents, it is very attractive as an oxidant for solution-phase reactions. Compared to other reagents, aqueous hydrogen peroxide is readily available, cheap, and is associated with easy handling, storage and transportation. This prompted us to compare the efficacy of H_2_O_2_ against other agents e.g. NaClO, KMnO_4_, *m*-CPBA, and the results are provided in Table 1.

**Table 1:** Effect of different oxidizing agents for the synthesis of **7a**. ^*^

Entry	Oxidant	Catalyst	Molar ratio (Sub:Oxid)	Solvent	Yield (%)
1	*m*-CPBA	(NH_4_)_6_Mo_7_O_24_	1:3	chloroform	28
2	KMnO_4_	-	1:2	glacial acetic acid	0
3	NaClO	-	1:4	water	0
4	H_2_O_2_	Al_2_O_3_	1:4	glacial acetic acid	45
5	H_2_O_2_	-	1:4	glacial acetic acid	48
6	H_2_O_2_	Na_2_WO_4_·2H_2_O	1:4	glacial acetic acid	55

* 0.43 mmol of intermediate **6a**, 12 mL of solvent, the reaction time for entries 1-3 was 70 min, for entries 4-6 was 40 min; all the reactions were conducted at 45 °C.

It could be easily observed that amongst all the oxidizing agents, H_2_O_2_ afforded the highest yield of the product (Table 1, Entries 4–6). Since some Lewis acid metal catalysts possessing vacant orbitals e.g. Sc(OTf)_3_ [23], Fe(III)-and Mn(III)-*meso*-tetraarylporphyrin [24], (NH_4_)_2_MoO_4_ [25] and methyltrioxorhenium(VII) (CH_3_ReO_3_, abbreviated as MTO) [26] are known to catalyze certain oxidative reactions, the role of catalysts Al_2_O_3_, (NH_4_)_2_MoO_4_, and Na_2_WO_4_·2H_2_O was studied in selected cases. The yield of sulfone was found to be considerably lower with the oxidant *m*-CPBA in the presence of the catalyst (NH_4_)_2_MoO_4_ (Entry 1) and practically negligible for the uncatalyzed reactions using oxidants KMnO_4_ and NaClO, respectively (Entries 2 and 3). When the oxidation with hydrogen peroxide was executed in the presence of catalysts Al_2_O_3_ and Na_2_WO_4_·2H_2_O, the later exhibited much superior activity compared to the former (Entries 4 and 6). This is indeed in line with the observation noted by Sato *et al.* [27] who reported an effective conversion of diaryl sulfides into sulfones by the oxidant hydrogen peroxide in the presence of the catalyst sodium tungstate. Having established the role of the oxidant H_2_O_2_ and the catalyst Na_2_WO_4_·2H_2_O, a systematic study of the effect of reaction parameters e.g., reaction temperature, time, solvent, molar ratio (substrate: oxidant) and the amount of catalyst was undertaken for optimization of the reaction. The results for the synthesis of **7a** are summarized in Table 2.

First, the effect of amount of the oxidant was studied. As the molar ratio of the reagents (substrate: hydrogen peroxide) was varied at 55 °C (Table 2, Entries 1–4), a maximum yield of 88% was noticed with a molar ratio of 1:6 (Table 2, Entry 2). On increasing the temperature from 45 °C to 50 °C and then 55 °C, the corresponding yields obtained were 77.0%, 84% and 88% (Table 2, Entries 5, 6, and 2) respectively. When the temperature was further increased to 60 °C and 70 °C, no improvement but rather a slight lowering in the yield (Table 2, Entries 7–8) was observed.

**Table 2:** Optimization of reaction conditions for the synthesis of **7a**.*

Entry	Molar ratio Sub:Oxi	T/°C	Time/min	Solvent	Yield (%)
1	1:5	55	50	glacial acetic acid	74
2	1:6	55	50	glacial acetic acid	88
3	1:7	55	50	glacial acetic acid	83
4	1:8	55	50	glacial acetic acid	64
5	1:6	45	50	glacial acetic acid	77
6	1:6	50	50	glacial acetic acid	84
7	1:6	60	50	glacial acetic acid	82
8	1:6	70	50	glacial acetic acid	77
9	1:6	55	40	glacial acetic acid	70
10	1:6	55	70	glacial acetic acid	84
11	1:6	55	50	acetonitrile	16
12	1:6	55	50	toluene	70
13	1:6	55	50	acetone	0
14	1:6	55	50	ethanol	0
15	1:6	55	50	DMF	41

^*^ Reaction conditions: 0.43 mmol of intermediate **6a**, 12 mL of solvent, oxidant was H_2_O_2_, catalyst was Na_2_WO_4_·2H_2_O.

Next, in order to obtain ideal reaction time, the reaction was also carried out for 40 min and 70 min (Entries 9 and 10), but the most suitable time was found to be 50 min (Entry 2). Amongst the different solvents examined, yields were found to be significantly lower in *N,N*-dimethylformamide (DMF), acetonitrile and toluene as compared to glacial acetic acid, while practically no product was observed in acetone and ethanol (Table 2, Entries 2 and 11–15). The ability of glacial acetic acid to serve both as a proton donor and a miscible cosolvent for organic/aqueous phase might account for this observation. Based on these results, the optimal conditions for the synthesis of sulfone are established with a molar ratio of (substrate:oxidant) 1:6 in glacial acetic acid at 55 °C for 50 min. As may be seen from Table 3, using optimal conditions, the compounds **7a**-**7i** were obtained in high yields (80–92%).

**Table 3:** Yields of compound **7** under optimized conditions.

Entry	**Compound**	R	Yield (%)
1	**7a**	benzyl	88
2	**7b**	4-fluorobenzyl	85
3	**7c**	4-methoxybenzyl	89
4	**7d**	4-bromobenzyl	88
5	**7e**	4-methylbenzyl	80
6	**7f**	2-fluorobenzyl	89
7	**7g**	3-methoxybenzyl	92
8	**7h**	allyl	89
9	**7i**	methyl	88

### 2.2. Crystal Structure Analysis of ***7g***

**Figure 1:**
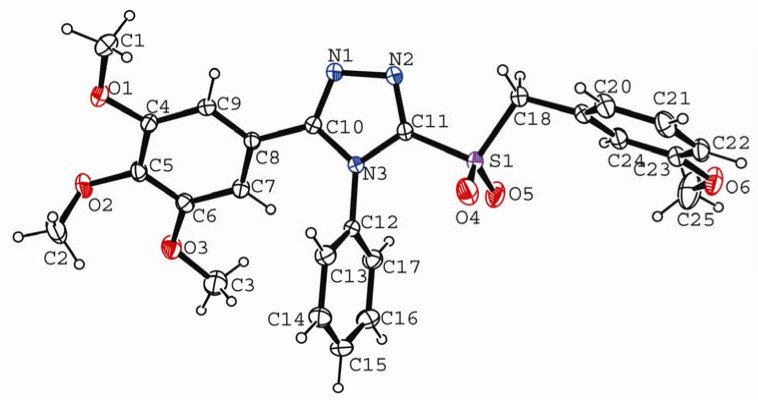
The molecular structure of **7g**.

**Figure 2:**
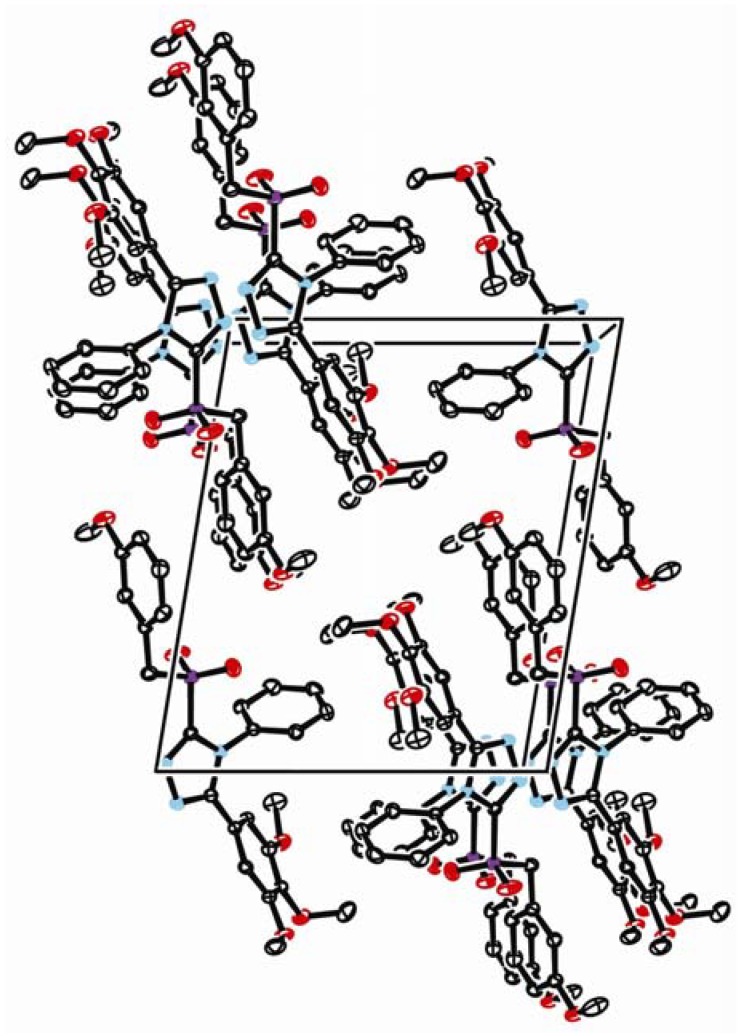
The packing of the molecule in crystal lattice of **7g**.

The crystal data and summary of data collection and structure refinement of **7g** are given in Table 4. Selected bond lengths and angles are given in Table 5. The molecular structure of compound **7g** is shown in Figure 1 and the packing of the molecule in crystal lattice is illustrated in Figure 2.

**Table 4:** Crystal data and summary of data collection and structure refinement.

Compound	C_25_H_25_N_3_O_6_S
Formula weight	495.54
Crystal system, Space group	Triclinic, p-1
a(nm)	0.74510(10)
b(nm)	1.17850(16)
c(nm)	1.39007(19)
*α*(°)	80.593(5)
*β*(°)	89.492(5)
*γ*(°)	88.471(5)
Volume(nm^3^)	1.2038(3)
Formula units	2
Calculated density(Mg/m^3^)	1.367
F(000)	520
Absorption correction	Semi-empirical frome quivalents
Refinement method	Full-matrix least-squares on F^2^
Data/restraints/parameters	4536/0/316
Goodness-of-fit on F^2^	1.062
Final R indices[I>2σ(*I*)]	R_1_ = 0.0426, ωR_1_ = 0.1103
Rindices(all data)	R_1_ = 0.0574, ωR_1_ = 0.1192

**Table 5:** Crystal data and summary of data collection and structure refinement.

Length	(nm)	Angle	(°)
C(1)-O(1)	0.1429(3)	C(7)-C(8)-C(10)	119.60(16)
C(4)-O(1)	0.1362(2)	N(1)-C(10)-N(3)	110.30(14)
C(4)-C(9)	0.1395(2)	N(1)-C(10)-C(8)	126.10(15)
C(5)-O(2)	0.1374(2)	N(3)-C(10)-C(8)	123.59(15)
C(6)-C(7)	0.1383(2)	N(2)-C(11)-N(3)	111.96(15)
C(8)-C(10)	0.1480(2)	N(2)-C(11)-S(1)	125.30(13)
C(10)-N(1)	0.1312(2)	N(3)-C(11)-S(1)	122.63(13)
C(10)-N(3)	0.1376(2)	C(17)-C(12)-C(13)	121.50(17)
C(11)-N(2)	0.1304(2)	C(17)-C(12)-N(3)	118.90(16)
C(11)-N(3)	0.1367(2)	C(13)-C(12)-N(3)	119.59(16)
C(11)-S(1)	0.1785(17)	C(19)-C(18)-S(1)	109.60(13)
C(12)-C(17)	0.1376(3)	C(20)-C(19)-C(24)	120.28(17)
C(12)-C(13)	0.1377(3)	C(20)-C(19)-C(18)	120.36(18)
C(12)-N(3)	0.1453(2)	C(24)-C(19)-C(18)	119.35(17)
C(16)-C(17)	0.1382(3)	C(10)-N(1)-N(2)	107.69(14)
C(18)-C(19)	0.1514(2)	C(11)-N(2)-N(1)	106.23(14)
C(18)-S(1)	0.1777(19)	C(11)-N(3)-C(10)	103.83(14)
C(19)-C(20)	0.1382(3)	C(11)-N(3)-C(12)	127.11(14)
C(20)-C(21)	0.1388(3)	C(10)-N(3)-C(12)	129.06(14)
C(22)-C(23)	0.1392(3)	O(4)-S(1)-O(5)	118.34(11)
C(23)-O(6)	0.1366(2)	O(4)-S(1)-C(18)	110.62(10)
C(25)-O(6)	0.1419(3)	O(5)-S(1)-C(18)	109.92(10)
N(1)-N(2)	0.1396(2)	O(4)-S(1)-C(11)	106.08(9)
O(4)-S(1)	0.1425(16)	O(5)-S(1)-C(11)	108.40(9)
O(5)-S(1)	0.1427(16)	C(18)-S(1)-C(11)	102.14(8)

From the bond length data it can be observed that the bond length of N(1)-N(2) is 0.1396 nm, which is shorter than the normal single N-N bond length (0.1450 nm). The bond lengths of 0.1376 nm and 0.1367 nm for N(3)-C(10) and N(3)-C(11), respectively, are shorter than normal single N-C bond length (0.1470 nm) and hence indicative of some double bond character. Again, the N(1)-C(10) (0.1312 nm) and the N(2)-C(11) (0.1304 nm) bond lengths are significantly closer to that of a typical C=N bond (0.134 nm). The N-C and N=C bonds near the phenyl ring are longer than the symmetrical bonds present near the sulfone presumably due to the conjugation of the phenyl with the triazole ring. The bond lengths observed in the 1,2,4-tirazole ring are in agreement with those found in other related studies [28,29]. The bond angle of C(10)-N(1)-N(2) is 107.69(14)° and C(11)-N(2)-N(1) is 106.23(14)°. The bond angle values further confirm the presence of delocalization in the triazole ring.

In the title compound, three phenyl rings (*p3*) are situated at different orientations with the triazole ring (*p1*). The dihedral angle between *p1* and the 3,4,5-trimethoxyphenyl moiety (*p2*) is 42.53°, *p1* and *p3* is 75.25°, *p1* and 4-methoxyphenyl (*p4*) is 79.29°. *p2 ,* *p3* and *p4* are torsional leading to a steady system. In the crystal structure, C-H⋯π supramolecular interactions occur between adjacent molecules with C(1)-H1B⋯*p4* angle of 155.30°, H1B⋯*p4* distance of 0.27375 nm, C(1)⋯*p4* distance of 0.3632(3) nm, C(3)-H3C⋯*p1* angle of 128.81°, H3C⋯*p1* distance of 0.30359 nm, C(3)⋯*p1* distance of 0.3714(3) nm, C(14)-H14⋯*p2* angle of 155.46°, H14⋯*p4* distance of 0.26964 nm and C(14)⋯*p2* distance of 0.3563(2) nm. In the solid state, the above mentioned hydrogen bonds that connect the molecules through a three dimensional network presumably stabilize the crystal structure.

## 3. Experimental

### 3.1. General

Unless otherwise stated, all the reagents and reactants were purchased from commercial suppliers; melting points were determined on a XT-4 binocular microscope (Beijing Tech Instrument Co., China) and are uncorrected; the ^1^H-NMR and ^13^C-NMR spectra were recorded on a JEOL ECX 500 NMR spectrometer at room temperature operating at 500 MHz for ^1^H-NMR and 125 MHz for ^13^C-NMR by using CDCl_3_ as the solvent and TMS as an internal standard; infrared spectra were recorded in KBr on a Bruker VECTOR 22 spectrometer; elemental analysis was performed on an Elemental Vario-III CHN analyzer. The course of the reactions was monitored by TLC; analytical TLC was performed on silica gel GF_254_; column chromatographic purification was carried out using silica gel. The carbothiamide (**4**) was prepared as described in the literature from gallic acid as the starting material through esterification and hydrazidation [30].

### 3.2. Preparation of 4-phenyl-5-(3,4,5-trimethoxyphenyl)-4H-1,2,4-triazole-3-thiol *(**5**) [16]*

A solution of compound **4** (2.39 g, 6.62 mmol) in 2 mol/L aqueous sodium hydroxide (5 mL, 10.0 mmol) was heated under reflux for 8 h. After cooling to room temperature, the solution was acidified with dilute hydrochloric acid. The precipitate was filtered off, washed with water, dried and recrystallized from ethanol to afford a white solid; yield 70.0%; m.p. 185–186 °C.

### 3.3. Preparation of 3-(substituted methylthio)-4-phenyl-5-(3,4,5-trimethoxyphenyl)-4H-1,2,4-triazoles ***6***

A mixture of 4-phenyl-5-(3,4,5-trimethoxyphenyl)-4H-1,2,4-triazole-3-thiol (**5**) (0.54 g, 1.57 mmol), 2.6% aqueous NaOH solution (1.57 mmol) and water (20 mL) was stirred at room temperature for 5 min; then the appropriate halides (1.57 mmol) and InCl_3_ (0.34 g, 0.157 mmol) were added and the solution was further stirred for an additional 5 h. The mixture was filtered and the resulting crude solid was washed with 5% Na_2_CO_3_ solution and distilled water, dried under vacuum, and recrystallized from methanol and water (v/v = 3:1) to afford the pure compounds **6**.

*3-(Benzylthio)-4-phenyl-5-(3,4,5-trimethoxyphenyl)-4H-1,2,4-triazole* (**6a**): This compound was obtained as a white solid; yield 88%; m.p. 136–138 °C (lit [16] 139–141 °C); ^1^H-NMR: *δ* 3.61 (s, 6H, 3,5-di-CH_3_O), 3.82 (s, 3H, 4-CH_3_O), 4.47 (s, 2H, CH_2_S), 6.63 (s, 2H, 3,4,5-trimethoxybenzyl-H), 7.14-7.49 (m, 10H, Ar-H); ^13^C-NMR: *δ* 37.43, 55.91, 60.97, 105.16, 121.75, 127.66, 127.81, 128.72, 129.31, 129.97, 130.06, 134.59, 136.57, 139.08, 152.53, 153.09, 154.61.

*3-(4-Fluorobenzylthio)-4-phenyl-5-(3,4,5-trimethoxyphenyl)-4H-1,2,4-triazole* (**6b**): white solid, yield 85%; m.p. 88–90 °C; IR (KBr cm^-1^): *ν* 1587, 1521, 1487, 1462, 1431 (C=N and C=C of rings), 698 (C-S-C); ^1^H-NMR: *δ* 3.59 (s, 6H, 3,5-di-CH_3_O), 3.82 (s, 3H, 4-CH_3_O), 4.43 (s, 2H, CH_2_S), 6.64 (s, 2H, 3,4,5-trimethoxybenzyl-H), 7.14-7.50 (m, 9H, Ar-H); ^13^C-NMR: *δ* 36.43, 55.91, 60.97, 105.25, 121.65, 127.61, 128.82, 130.01, 130.07, 130.68, 133.64, 134.53, 135.39, 139.22, 152.03, 153.11, 154.71; Anal. Calcd. for C_24_H_22_FN_3_O_3_S(451.51): C 63.84%, H 4.91%, N 9.31%. Found: C 63.68%, H 4.72%, N 9.26%.

*3-(4-Methoxybenzylthio)-4-phenyl-5-(3,4,5-trimethoxyphenyl)-4H-1,2,4-triazole* (**6c**): white solid, yield 94%; m.p. 135-136 °C (lit [16] 140–142 °C); ^1^H-NMR: *δ* 3.61 (s, 6H, 3,5-di-CH_3_O), 3.71 (s, 3H, 4-CH_3_O of 4-methoxybenzyl), 3.82 (s, 3H, 4-CH_3_O), 4.50 (s, 2H, CH_2_S), 6.64 (s, 2H, 3,4,5-trimethoxy-benzyl-H), 7.28-7.56 (m, 9H, Ar-H); ^13^C-NMR: *δ* 35.33, 55.31, 55.87, 55.94, 105.25, 113.62, 114.72, 121.76, 128.83, 129.71, 130.02, 134.67, 138.09, 139.12, 152.41, 153.19, 154.65, 159.71;

*3-(4-Bromobenzylthio)-4-phenyl-5-(3,4,5-trimethoxyphenyl)-4H-1,2,4-triazole* (**6d**): white solid, yield 88%; m.p. 165–167 °C; IR (KB, cm^-1^): *ν* 1587, 1508, 1498, 1463, 1425 (C=N and C=C of rings), 705 (C-S-C); ^1^H-NMR: *δ* 3.60 (s, 6H, 3,5-di-CH_3_O), 3.82 (s, 3H, 4-CH_3_O), 4.42 (s, 2H, CH_2_S), 6.63 (s, 2H, 3,4,5-trimethoxybenzyl-H), 7.13–7.50 (m, 9H, Ar-H); ^13^C-NMR: *δ* 36.49, 55.91, 60.97, 105.26, 121.64, 121.76, 127.61, 130.01, 130.07, 131.02, 131.79, 134.53, 135.93, 139.24, 152.09, 153.11, 154.72; Calcd. for C_24_H_22_BrN_3_O_3_S(512.42): C 56.25%, H 4.33%, N 8.20%. Found: C 56.16%, H 4.39%, N 8.30%.

*3-(4-Methylbenzylthio)-4-phenyl-5-(3,4,5-trimethoxyphenyl)-4H-1,2,4-triazole* (**6e**): white solid, yield 91.2%; m.p. 150–152 °C; IR (KBr cm^-1^): *ν* 1587, 1512, 1487, 1458, 1429 (C=N and C=C of rings), 705 (C-S-C); ^1^H-NMR: *δ* 2.31 (s, 3H, 4-CH_3_ of 4-methylbenzyl), *δ*: 3.60 (s, 6H, 3,5-di-CH_3_O), 3.82 (s, 3H, 4-CH_3_O), 4.45 (s, 2H, CH_2_S)，6.64 (s, 2H, 3,4,5-trimethoxybenzyl-H), 7.08-7.48 (m, 9H, Ar-H); ^13^C-NMR: *δ* 21.23, 37.10, 55.91, 60.97, 105.27, 121.80, 127.68, 129.22, 129.40, 129.90, 130.01, 133.43, 134.65, 137.59, 139.15, 152.71, 153.09, 154.56; Anal. Calcd. for C_25_H_25_N_3_O_3_S (447.55): C 67.09%, H 5.63%, N 9.39%. Found: C 67.20%, H 5.48%, N 9.42%.

*3-(2-Fluorobenzylthio)-4-phenyl-5-(3,4,5-trimethoxyphenyl)-4H-1,2,4-triazole* (**6f**): white solid, yield 89%; m.p. 88–90 °C; IR (KBr cm^-1^): *ν* 1587, 1521, 1487, 1462, 1431 (C=N and C=C of rings), 698 (C-S-C); ^1^H-NMR: *δ* 3.60 (s, 6H, 3,5-di-CH_3_O), 3.82 (s, 3H, 4-CH_3_O), 4.51 (s, 2H, CH_2_S), 6.64 (s, 2H, 3,4,5-trimethoxybenzyl-H), 7.00-7.51 (m, 9H, Ar-H); ^13^C-NMR: *δ* 30.47, 55.91, 60.97, 105.26, 115.63, 121.73, 124.12, 124.29, 127.64, 129.99, 130.06, 131.65, 134.55, 139.20, 152.34, 153.10, 154.71, 160.10, 162.07; Anal. Calcd. for C_24_H_22_FN_3_O_3_S (451.51): C 63.84%, H 4.91%, N 9.31%. Found: C 63,68%, H 4.72%, N 9.26%.

*3-(3-Methoxybenzylthio)-4-phenyl-5-(3,4,5-trimethoxyphenyl)-4H-1,2,4-triazole* (**6g**): white solid, yield 78.0%; m.p. 140–141 °C (lit [16] 145–147 °C); ^1^H-NMR: *δ* 3.59 (s, 6H, 3,5-di-CH_3_O), 3.74 (s, 3H, 3-CH_3_O of 3-methoxybenzyl), 3.80 (s, 3H, 4-CH_3_O), 4.43 (s, 2H, CH_2_S), 6.64 (s, 2H, 3,4,5-trimethoxy-benzyl-H), 7.28-7.48 (m, 9H, Ar-H); ^13^C-NMR: *δ* 37.43, 55.32, 55.87, 55.90, 105.23, 113.69, 114.72, 121.76, 127.63, 129.94, 130.02, 134.57, 138.00, 139.12, 152.45, 153.09, 154.65, 159.79.

*3-(Allylthio)-4-phenyl-5-(3,4,5-trimethoxyphenyl)-4H-1,2,4-triazole* (**6h**): white solid, yield 82.0%; m.p. 97–98 °C (lit [16] 102–104 °C); ^1^H-NMR: *δ* 3.60 (s, 6H, 3,5-di-CH_3_O), 3.82 (s, 3H, 4-CH_3_O), 3.91 (s, 2H, CH_2_S), 5.14-5.32 (m, 2H, C=CH_2_), 5.96–5.60 (m, 1H, HC=C), 6.64 (s, 2H, 3,4,5-trimethoxybenzyl-H), 7.27–7.56 (m, 5H, Ar-H); ^13^C-NMR: *δ* 35.56, 55.88, 61.03, 105.23, 119.22, 121.76, 127.74, 130.00, 130.11, 132.65, 134.66, 139.07, 152.30, 153.05, 154.67.

*3-(Methylthio)-4-phenyl-5-(3,4,5-trimethoxyphenyl)-4H-1,2,4-triazole* (**6i**): white solid, yield 76.0%. mp 102–104 °C (lit [16] 114–116 °C); ^1^H-NMR: *δ* 2.73 (s, 3H, CH_3_S), 3.60 (s, 6H, 3,5-di-CH_3_O), 3.82 (s, 3H, 4-CH_3_O), 6.64 (s, 2H, 3,4,5-trimethoxybenzyl-H), 7.27-7.55 (m, 5H, Ar-H); ^13^C-NMR: *δ* 14.86, 55.94, 61.01, 105.26, 121.80, 127.55, 130.05, 130.14, 134.65, 139.08, 153.08, 153.83, 154.77.

### 3.4. General procedure for the preparation of title compounds ***7***

To a three-necked 100 mL flask equipped with a magnetic stirrer was added 3-(substituted methylthio)-4-phenyl-5-(3,4,5-trimethoxyphenyl)-4*H*-1,2,4-triazole (0.43 mmol), acetic acid (10 mL) and Na_2_WO_4_·2H_2_O (0.007 g, 0.022 mmol). The resulting mixture was stirred for 5 min, and then 30% H_2_O_2_ (0.29 g, 2.58 mmol) was slowly added into the system, heated to 55 °C and the reaction was continued for 50 min. After cooling to room temperature, the mixture was neutralized by 5% sodium hydroxide to a pH of 7.0, extracted with chloroform (3 × 30 mL), dried over anhydrous magnesium sulfate, and separated on silica column with ethyl acetate /petroleum ether (v/v = 1:3) to give pure products **7**.

*3-(Benzylsulfonyl)-4-phenyl-5-(3,4,5-trimethoxyphenyl)-4H-1,2,4-triazole* (**7a**): white solid, yield 88.6%; m.p. 152–153 °C; IR (KBr cm^-1^): *ν* 1587, 1519, 1496, 1473, 1458 (C=N and C=C of rings), 1342, 1147 (SO_2_); ^1^H-NMR: *δ* 3.59 (s, 6H, 3,5-di-CH_3_O), 3.82 (s, 3H, 4-CH_3_O), 4.86 (s, 2H, CH_2_SO_2_), 6.62 (s, 2H, 3,4,5-trimethoxybenzyl-H), 6.99–7.49 (m, 10H, Ar-H); ^13^C-NMR: *δ* 55.91, 60.90, 61.73, 105.88, 120.00, 125.95, 127.63, 128.92, 129.34, 129.58, 130.47, 131.76, 133.35, 139.92, 152.23, 153.11, 155.82; Anal. Calcd. for C_24_H_23_N_3_O_5_S (465.52): C 61.92%, H 4.98%, N 9.03%. Found: C 62.01%, H 4.51%, N 9.05%.

*3-(4-Fluorobenzylsulfonyl)-4-phenyl-5-(3,4,5-trimethoxyphenyl)-4H-1,2,4-triazole* (**7b**): white solid, yield 85.0%; m.p. 152–153 °C; IR (KBr cm^-1^): *ν* 1585, 1521, 1496, 1473, 1458 (C=N and C=C of rings), 1338, 1158 (SO_2_); ^1^H-NMR: *δ* 3.60 (s, 6H, 3,5-di-CH_3_O), 3.83 (s, 3H, 4-CH_3_O), 4.87 (s, 2H, CH_2_SO_2_), 6.64 (s, 2H, 3,4,5-trimethoxybenzyl-H), 7.15-7.52 (m, 9H, Ar-H); ^13^C-NMR: *δ* 56.00, 60.76, 61.00, 105.97, 119.93, 124.58, 127.69, 129.20, 129.81, 130.68, 133.15, 133.33, 135.80, 139.94, 152.27, 153.23, 156.05; Anal. Calcd. for C_24_H_22_FN_3_O_5_S (483.51): C 59.62%, H 4.59%, N 8.69%. Found: C 59.91%, H 4.96%, N 8.86%.

*3-(4-Methoxybenzylsulfonyl)-4-phenyl-5-(3,4,5-trimethoxyphenyl)-4H-1,2,4-triazole* (**7c**): white solid, yield 89.0%; m.p. 182–184 °C; IR (KBr cm^-1^): *ν* 1585, 1517, 1496, 1473, 1458 (C=N and C=C of rings), 1334, 1147 (SO_2_); ^1^H-NMR: *δ* 3.61 (s, 6H, 3,5-di-CH_3_O), 3.71 (s, 3H, 4-CH_3_O of 4-methoxybenzyl), 3.87 (s, 3H, 4-CH_3_O), 4.82 (s, 2H, CH_2_SO_2_), 6.64 (s, 2H, 3,4,5-trimethoxybenzyl-H), 7.04-7.51 (m, 9H, Ar-H); ^13^C-NMR: *δ* 55.80, 60.45, 60.92, 105.94, 115.60, 120.85, 122.90, 127.91, 129.64, 130.04, 130.98, 131.70, 133.01, 135.01, 139.80, 153.26, 153.41, 160.64; Anal. Calcd. for C_25_H_25_N_3_O_6_S (495.55): C 60.59%, H 5.08%, N 8.48%. Found: C 60.70%, H 5.04%, N 8.55%.

*3-(4-Bromobenzylsulfonyl)-4-phenyl-5-(3,4,5-trimethoxyphenyl)-4H-1,2,4-triazole* (**7d**): white solid, yield 88.0%; m.p. 172–173 °C; IR (KBr cm^-1^): *ν* 1585, 1520, 1498, 1479, 1458 (C=N and C=C of rings), 1332, 1145(SO_2_); ^1^H-NMR: *δ* 3.60 (s, 6H, 3,5-di-CH_3_O), 3.83 (s, 3H, 4-CH_3_O), 4.85 (s, 2H, CH_2_SO_2_), 6.64 (s, 2H, 3,4,5-trimethoxybenzyl-H), 7.14-7.52 (m, 9H, Ar-H); ^13^C-NMR: *δ* 55.91, 60.76, 60.91, 105.90, 119.84, 123.94, 125.04, 127.59, 129.72, 130.60, 132.10, 133.24, 133.35, 139.88, 152.16, 153.15, 155.98; Anal. Calcd. for C_24_H_22_BrN_3_O_5_S (544.42): C 52.95%, H 4.07%, N 7.72%. Found: C 53.35%, H 4.29%, N 7.52%.

*3-(4-Methylbenzylsulfonyl)-4-phenyl-5-(3,4,5-trimethoxyphenyl)-4H-1,2,4-triazole* (**7e**): white solid, yield 80.2%; m.p. 181–183 °C; IR (KBr cm^-1^): *ν* 1587, 1514, 1498, 1475, 1454 (C=N and C=C of rings), 1334, 1143 (SO_2_); ^1^H-NMR: *δ* 2.35 (s, 3H, 4-CH_3_ of 4-methylbenzyl), 3.60 (s, 6H, 3,5-di-CH_3_O), 3.82 (s, 3H, 4-CH_3_O), 4.83 (s, 2H, CH_2_SO_2_), 6.63 (s, 2H, 3,4,5-trimethoxybenzyl-H), 7.04-7.51 (m, 9H, Ar-H); ^13^C-NMR: *δ* 21.35, 55.99, 61.00, 60.99, 61.48, 105.95, 120.14, 122.83, 127.75, 129.68, 130.53, 131.70, 133.48, 139.50, 140.04, 152.44, 153.18, 155.88; Anal. Calcd. for C_25_H_25_N_3_O_5_S (479.55): C 62.61%, H 5.25%, N 8.76%; Found: C 62.61%, H 4.76%, N 8.52%.

*3-(2-Fluorobenzylsulfonyl)-4-phenyl-5-(3,4,5-trimethoxyphenyl)-4H-1,2,4-triazole* (**7f**): white solid. yield 89.0%; m.p. 153–154 °C; IR (KBr cm^-1^): *ν* 1587, 1519, 1496, 1477, 1460 (C=N and C=C of rings), 1323, 1153 (SO_2_); ^1^H-NMR: *δ* 3.61 (s, 6H, 3,5-di-CH_3_O), 3.83 (s, 3H, 4-CH_3_O), 4.97 (s, 2H, CH_2_SO_2_), 6.67 (s, 2H, 3,4,5-trimethoxybenzyl-H), 7.09-7.54 (m, 9H, Ar-H); ^13^C-NMR: *δ* 54.94, 56.02, 60.99, 106.04, 113.71, 115.95, 116.12, 120.05, 124.62, 127.79, 130.70, 131.68, 133.44, 139.78, 152.52, 153.23, 156.16, 160.98, 162.97; Anal. Calcd. for C_24_H_22_FN_3_O_5_S (483.51): C 59.62%, H 4.59%, N 8.69%. Found: C 59.50%, H 4.54%, N 8.30%.

*3-(3-Methoxybenzylsulfonyl)-4-phenyl-5-(3,4,5-trimethoxyphenyl)-4H-1,2,4-triazole* (**7g**): white solid, yield 92.8%; m.p. 153–155 °C; IR (KBr cm^-1^): *ν* 1598, 1514, 1492, 1471, 1460(C=N and C=C of rings), 1321, 1124(SO_2_); ^1^H NMR (CDCl_3_, 500 MHz): *δ* 3.59 (s, 6H, 3,5-di-CH_3_O), 3.73 (s, 3H, 3-CH_3_O of 3- methoxybenzyl), 3.82 (d, 3H, 4-CH_3_O), 4.81 (s, 2H, CH_2_SO_2_)，6.62 (s, 2H, 3,4,5-trimethoxy benzyl-H), 6.83-7.50 (m, 9H, Ar-H); ^13^C NMR (CDCl_3_, 125 MHz): *δ* 55.41, 55.99, 60.98, 61.96, 105.96, 115.76, 116.49, 120.10, 124.05, 127.74, 129.60, 129.97, 130.51, 133.42, 140.04, 153.18, 155.94, 159.96; Anal. Calcd. for C_25_H_25_N_3_O_6_S (495.55): C 60.59%, H 5.08%, N 8.48%. Found: C 60.56%, H 5.43%, N 8.87%.

*3-(Allylsulfonyl)-4-phenyl-5-(3,4,5-trimethoxyphenyl)-4H-1,2,4-triazole* (**7h**): white solid, yield 89.5%; m.p. 132–134 °C; IR (KBr cm^-1^): *ν* 1589, 1518, 1496, 1479, 1456 (C=N and C=C of rings), 1325, 1145 (SO_2_); ^1^H-NMR: *δ* 3.591 (s, 6H, 3,5-di-CH_3_O), 3.82 (s, 3H, 4-CH_3_O), 4.33 (s, 2H, CH_2_SO_2_), 5.51-5.55 (m, 2H, C=CH_2_), 5.89-5.95 (m, 1H, HC=C), 6.65 (s, 2H, 3,4,5-trimethoxybenzyl-H), 7.42-7.56 (m, 5H, Ar-H); ^13^C-NMR: *δ* 55.98, 59.52, 61.00, 105.94, 120.05, 122.90, 126.81, 127.87, 129.97, 130.79, 133.45, 140.00, 152.50, 153.19, 156.01; Anal. Calcd. for C_20_H_21_N_3_O_5_S (415.46): C 57.82%, H 5.09%, N 10.11%. Found: C 57.52%, H 5.38%, N 10.45%.

*3-(Methylsulfonyl)-4-phenyl-5-(3,4,5-trimethoxyphenyl)-4H-1,2,4-triazole* (**7i**): white solid, yield 80.2%; m.p. 141–143 °C, IR (KBr cm^-1^): *ν* 1587, 1514, 1498, 1475, 1454 (C=N and C=C of rings), 1334, 1143 (SO_2_), ^1^H-NMR: *δ* 3.49 (s, 3H, CH_3_-SO_2_), 3.59 (s, 6H, 3,5-di-CH_3_O), 3.81 (s, 3H, 4-CH_3_O), 6.64 (s, 2H, 3,4,5-trimethoxybenzyl-H), 7.41-7.54 (m, 5H, Ar-H); ^13^C-NMR: *δ* 43.02, 55.99, 60.97, 106.01, 119.99, 127.81, 130.03, 130.81, 133.40, 140.09, 153.22, 153.60, 156.22; Anal Calcd. for C_18_H_19_N_3_O_5_S (389.43): C 55.52%, H 4.92%, N 10.79%. Found: C 55.32%, H 4.61%, N 10.84%.

### 3.5. X-ray structure determination of ***7g***

A crystal of the title compound with dimensions of 0.20 mm × 0.22 mm × 0.18 mm was mounted on a Bruker SM ART Apex area-detector diffractometer with a graphite-monochromated Mo-*K*a radiation(*λ* = 0.071073 nm) by using an *φ-ω* scan mode at 293(2) K in the range of 1.48 ≤ θ ≤ 25.99. A total of 12566 reflections were collected, of which 4536 were independent (*R*int = 0.0318) and 3543 were observed with *I* > 2σ(*I*). The calculations were performed with SHELXS-97 program [31] and the empirical absorption corrections were applied to all intensity data. The non-hydrogen atoms were refined anisotropically. The hydrogen atoms were determined with theoretical calculations and refined isotropically. The final full-matrix least-squares refinement gave *R* = 0.0426 and ω*R* = 0.1103 (ω = l/[*σ*^2^(*Fo^2^*) + (0.0607*P*)^2^ + 0.0212*P*], where *P* = (*Fo^2^ + 2Fc^2^*)/3). S = 1.033, (Δ*σ*)_max_/(e.nm^-3^) = 174, (Δ*σ*)_min_/(e.nm^-3^) = -353. Crystallographic data (excluding structure factors) for the structure have been deposited with the Cambridge Crystallographic Data Center as supplementary publication No. CCDC 703877. These data can be obtained free of charge from the CCDC *via* www.ccdc.cam.ac.uk/ data_request/cif.

## 4. Conclusions

In the present study, a mild and effective method for the preparation of novel sulfone derivatives containing 1,2,4-triazole moieties was undertaken using gallic acid as the starting material. The key step of the oxidation from thioether to the corresponding sulfone was optimized. The method has some salient features such as faster reaction rates, high yields and environmental friendliness. The synthesized compounds were characterized by spectral data (^1^H-NMR, ^13^C-NMR, IR) and elemental analysis. Furthermore, 3-(3-methoxybenzylsulfonyl)-4-phenyl-5-(3,4,5-trimethoxyphenyl)-4*H*-1,2,4-triazole (**7g**) was investigated by X-ray crystallographic analysis. In the solid state, a three dimensional hydrogen bond network within the molecule presumably imparts stability to the crystal lattice.
